# A Case of Severe Tricuspid Valve Regurgitation in a Patient Undergoing Orthotopic Liver Transplantation: Whether to Proceed, or Not

**DOI:** 10.7759/cureus.24119

**Published:** 2022-04-13

**Authors:** Justin Mitchell, Caroline E Tybout, Leonid Gorelik, Sujatha P Bhandary, Antolin S Flores

**Affiliations:** 1 Anesthesiology, The Ohio State University College of Medicine, Columbus, USA; 2 Anesthesiology, The Ohio State University Wexner Medical Center, Columbus, USA; 3 Anesthesiology, Emory University School of Medicine, Columbus, USA

**Keywords:** preoperative evaluation, right heart failure, graft survival, transesophageal echocardiography, congestive hepatopathy, tricuspid regurgitation, liver transplantation

## Abstract

A 38-year-old male presented for orthotopic liver transplantation complicated by new-onset torrential tricuspid regurgitation before incision. Subclinical volume overload and functional tricuspid regurgitation created a challenging scenario in which the benefits of expeditious transplant were weighed against the risks of allograft congestion and failure. Intraoperative transesophageal echocardiography proved critical in diagnosing severe tricuspid regurgitation and guided clinical decision making. In this article, we describe the intraoperative presentation of acutely elevated right heart pressures and the subsequent management of this patient prior to ultimately successful liver transplantation.

## Introduction

The pathophysiology of end-stage liver disease can cause significant hemodynamic and cardiovascular disturbances [1]. These complex changes occur because of diseased liver parenchyma and increased portal system pressures which lead to fluid retention and volume overload, potentially precipitating to high output heart failure in the perioperative setting [2]. Distention of the right ventricle from excessive intravascular volume causes dilatation of the tricuspid valve apparatus and functional tricuspid regurgitation (TR). Elevated preoperative right ventricular systolic pressure (RVSP) and TR can threaten graft survival through congestive hepatopathy and contribute to postoperative mortality [3]. Herein, we report a case of new-onset severe TR, emphasizing the importance of delineating volume status, careful intraoperative transesophageal echocardiography (TEE) monitoring, and avoiding potential complications of vascular congestion on liver allografts in liver transplant recipients.

## Case presentation

A 38-year-old male with decompensated alcoholic cirrhosis complicated by spontaneous bacterial peritonitis and type 1 hepatorenal syndrome (HRS) presented for orthotopic liver transplantation (OLT). On initial evaluation, he had prerenal azotemia, demonstrated by a fractional excretion of sodium (FENa) of <1% with an anion gap metabolic acidosis, secondary to diarrhea. The patient was fluid resuscitated after excluding portopulmonary hypertension (PPHTN) and acute heart failure with echocardiography. Despite this, the patient remained oliguric, hyperkalemic, and increasingly acidotic, necessitating continuous renal replacement therapy (CRRT) while awaiting liver transplantation.

On evaluation for liver transplantation, the patient’s vital signs were within normal limits with no overt findings of fluid overload on physical exam. His model for end-stage liver disease (MELD)-Na score was 45 points, and an echocardiogram demonstrated trace TR, an RVSP of 33 mmHg, and a right-to-left interatrial shunt. The patient had normal left ventricular (LV) size and function, with an ejection fraction of 60%.

**Figure 1:**
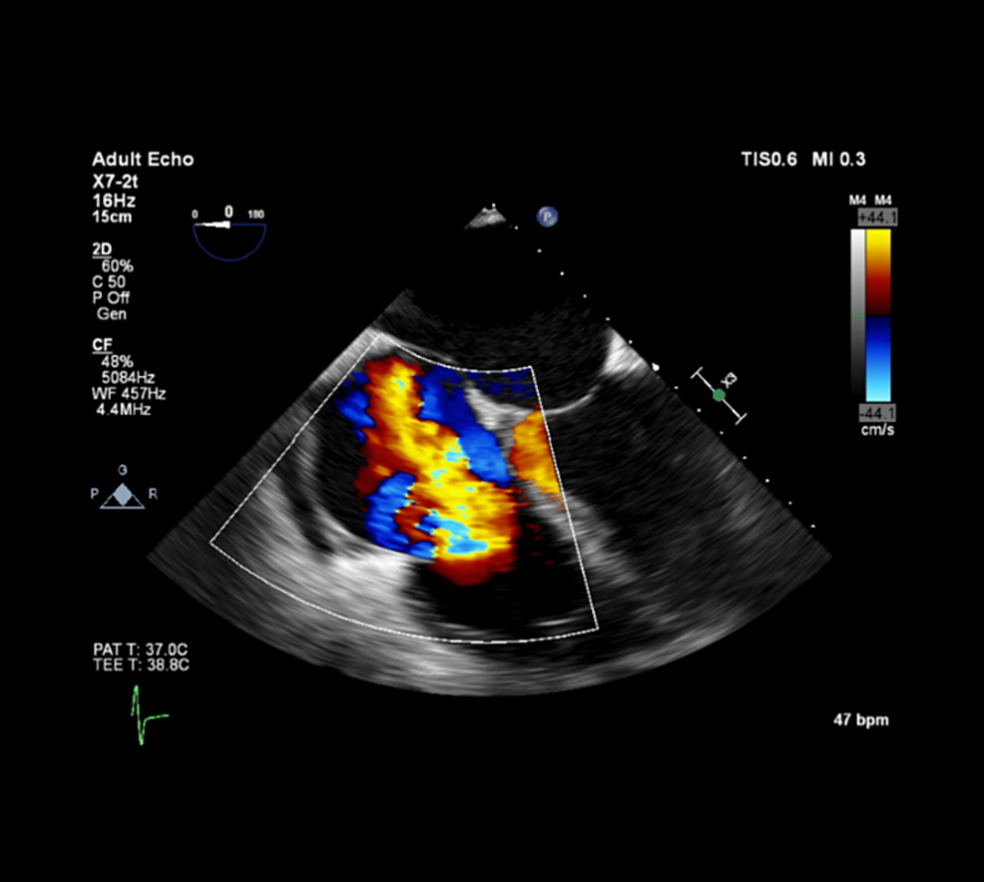
Transesophageal echocardiography (TEE) image demonstrating severe tricuspid regurgitation (TR) on initial presentation for liver transplantation (mid-esophageal 4-chamber view).

On the day of the transplant, intraoperative pulmonary arterial catheterization revealed pulmonary artery pressures (PAP) of 120s-140s/70s [mean pulmonary artery pressures (MPAP) 60s-90s], central venous pressures (CVP) of 40s-80s, and mean arterial pressures (MAPs) in the 50s. Intraoperative TEE before incision surprisingly revealed severely elevated right-sided pressures with leftward bowing of the intra-atrial septum. New severe TR was present with overall preserved right ventricular function (Figure 1). The findings were discussed within a multidisciplinary team of transplant surgeons and anesthesiologists, and a decision was made to abort the surgery. Like many transplant centers, our institution interprets an MPAP of >50 mmHg as a cut-off for cancelation, as MPAP > 35 mmHg has been associated with adverse outcomes [4].

**Figure 2:**
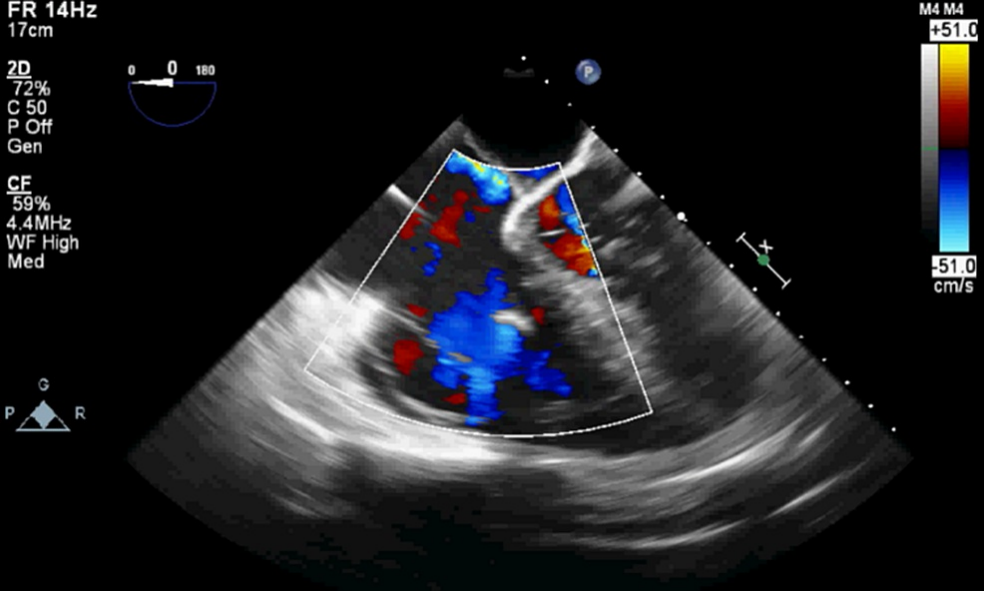
Transesophageal echocardiography (TEE) image demonstrating almost no tricuspid regurgitation (TR) on second presentation for liver transplantation after aggressive volume removal (mid-esophageal 4-chamber view).

The patient was then transported to the intensive care unit, where his transpulmonary gradient was calculated to be <12 mmHg, once again excluding right heart failure. The critical care team pursued aggressive diuresis with CRRT. A repeat echocardiogram after four days of CRRT showed improvement of right ventricular size, with a reduction in the TR severity and a bidirectional shunt across a patent foramen ovale (PFO). Furthermore, the patient exhibited no clinical signs suggestive of severe TR including fatigue, dyspnea, or lower extremity edema. The patient was re-listed for liver transplantation and successfully underwent surgery 10 days later. The perioperative TEE during that time showed mild TR and flow only going left-to-right across the PFO (Figure 2). During the postoperative period, the patient remained hemodynamically stable with downtrending liver function tests (LFTs) - discharge LFTs - aspartate transaminase (AST) 24, alanine transaminase (ALT) 87, albumin 3.3, alkaline phosphatase 93. There were no significant electrolyte, metabolic, or coagulation abnormalities during his postoperative course and he was subsequently discharged on postoperative day 8 without complication. He was readmitted seven days later with abdominal pain and found to have a biliary stricture that was treated with a sphincterotomy and biliary stent by gastroenterology, otherwise the transplanted liver was functioning normally.

## Discussion

This case report highlights the importance of intraoperative vigilance when assessing the right ventricle and volume status to avoid potentially disastrous complications during liver transplantation. The limitations of clinical examination and the advantages of point-of-care ultrasound to evaluate for signs of volume overload have been well described [5-7]. In our patient with masked hypervolemia and prerenal azotemia, the decision to fluid resuscitate was weighed carefully against the risk of exacerbating any underlying right heart disease or PPHTN. The delicate interplay between diuresis and fluid overload involves multiple organ systems and proves difficult in patients with severe comorbidities. Intraoperative TEE was imperative to ascertaining the hemodynamic status of the patient and guiding clinical decision-making. As stated previously, cardiac stress testing just one week before the attempted operation showed only trace TR, mildly elevated RVSP, sustained ejection fraction, and the patient exhibited no signs of significant pulmonary hypertension on the presentation for surgery.

However, during intraoperative TEE imaging, new-onset torrential TR was identified with elevated PAP. In the absence of intraoperative TEE, any significant improvements in MPAP could have misled one to believe the elevation was a temporary manifestation of Trendelenburg positioning and/or fluctuating depth of anesthesia in the setting of intravascular volume overload. If the surgery had proceeded, the effects of cross-clamp release during the reperfusion period on the cardiopulmonary circulation may have been catastrophic, as the stress of reperfusion on an already compromised right ventricle would have likely resulted in cardiopulmonary failure, graft congestion and failure, and death [8-12].

A retrospective analysis of 64 OLT recipients showed a statistically significant difference in patient mortality in those with an elevated systolic PAP, increased postoperative right ventricular diameter, and lower tricuspid annular plane systolic excursion obtained by echocardiography [13]. The presence of new-onset TR on TEE examination serves as an additional “red flag” indicative of acute right heart dysfunction. In a retrospective cohort study of 397 adult liver transplant recipients, TR graded greater than mild was associated with an increased risk of post-transplant graft failure nearly four-fold [14] and postoperative mortality by 70% [15] to 400% [14]. This may reflect the inability of the right ventricle to accommodate the increased central blood volume. Likewise, another retrospective analysis of 84 living donor liver transplant recipients demonstrated that subclinical elevated TR pressure gradient (≥25 mmHg) was strongly associated with decreased three-month and one-year survival post-liver transplant [16].

In summary, our case highlights the importance of cardiac monitoring during liver transplantation and identifying instances where a patient has greater than mild TR. This patient’s rapid decline in cardiac function, detected by intraoperative TEE, demonstrates the acuity in which severe functional TR can present. Across the OLT patient population, unexpected cardiac findings during intraoperative TEE evaluation have changed the course of management in about 1 in 10 of these patients [17]. Congruently, real-time evidence of severe TR and elevated right-sided pressures on intraoperative TEE provided considerable guidance in our decision to abort the case [18].

## Conclusions

Liver transplantation in patients with severe TR carries a high perioperative mortality and morbidity. TEE has been shown in combination with standard invasive hemodynamic monitoring to help guide intraoperative management of cardiopulmonary derangements and even help delineate a diagnosis before proceeding with transplantation. In our case, TEE served as an effective and reliable diagnostic tool when PAC monitoring alone provided inconclusive and variable results. The clinical value of TEE in combination with collaborative decision-making to delay rather than proceed with liver transplantation likely provided a better outcome for the patient while preventing possible vascular congestion and graft failure.
